# The relationship among idiosyncratic deals, psychological empowerment, and internal locus of control: A moderated mediation model

**DOI:** 10.3389/fpsyg.2022.923874

**Published:** 2022-11-16

**Authors:** Muhammad Shahid Shams, Tang Swee Mei, Zurina Adnan, Murtaza Masud Niazi, Kaleemullah Khan

**Affiliations:** ^1^Department of Business Administration, Kardan University, Kabul, Afghanistan; ^2^School of Business Management, Universiti Utara Malaysia, Sintok, Malaysia

**Keywords:** idiosyncratic deals, internal locus of control, psychological empowerment, work engagement, higher education

## Abstract

Building upon the job demands–resources (JD-R) theory, this research offers an in-depth exploration of the mechanisms by which idiosyncratic deals (I-deals), such as personalized work arrangements, can enhance academics’ psychological empowerment (PE) and hence affect their work engagement. This study’s purpose was to investigate whether PE mediates the relationships between task and work responsibilities I-deals, flexibility I-deals, and work engagement among academics in higher education and whether the mediating effects are moderated by academics’ internal locus of control. Using an online platform, the survey questionnaire was sent to 650 academics working in higher education. The results reveal that task and work responsibilities I-deals and flexibility I-deals, are positively associated with the academics’ work engagement and that PE mediates those relationships. Additionally, the internal locus of control strengthens the positive relationship between task and work responsibilities I-deals and PE, and it enhances the indirect effect of task and work responsibilities I-deals on academics’ work engagement through PE. Though, this study did not find the moderating effect of internal locus of control on the flexibility I-deals–PE relationship; however, the results indicate that internal locus of control boosts the indirect effect of flexibility I-deals on academics’ work engagement through PE.

## Introduction

The competitive and dynamic environment has created challenges for higher education institutions (HEIs) worldwide to develop and maintain engaged academics, who are involved in, enthusiastic about, and committed to their work and positively contribute to their organization (Schaufeli et al., 2002; Schaufeli and Bakker, 2010; Joo et al., 2016). Work engagement (WE) refers to the degree to which people feel energized and enthusiastic regarding their work. It is characterized by vigor, dedication, and absorption (Schaufeli and Bakker, 2009). Engaged workers are full of energy (vigor), strongly involved in their work (dedication), and often fully concentrated and happily engrossed in their work activities (absorption). HEIs, being knowledge-intensive organizations, largely depend on their academics’ commitment and engagement (Nazir and Islam, 2017; Aboramadan et al., 2020). This is because academics with high WE tend to reflect higher levels of psychological commitment and loyalty (Lovakov, 2016), ensuring students’ success by well equipping them with skills necessary to meet corporate requirements as well as the attainment of educational objectives (Hajdarpasic et al., 2015; Raina and Khatri, 2015; González-Rico et al., 2018; Wasilowski, 2018), quality of academic contributions, research publications, and success of the HEIs (Gloria and Steinhardt, 2017; Christensen et al., 2020). Therefore, the ability of HEIs to develop and maintain engaged academics becomes even more essential.

Higher education institutions are complex organizations with scarce resources with the priority of keeping their academics engaged all the time. However, achieving a team of engaged academics is not that easy as it requires a high level of energy and resources (Macey and Schneider, 2008). Relatedly, psychological empowerment (PE), defined as how empowered an employee feels (Spreitzer, 1995), has been documented as an essential motivational resource that enables workers to be more engaged in the workplace (Ugwu et al., 2014). According to Spreitzer (1995), PE represents the psychological states of the employees (subordinates) resulting from empowering practices in the workplace, including the four dimensions of employee perceptions: *competence* (the belief of an employee of his/her capabilities to accomplish a task), *impact* (an employee’s influence over the organization’s outcomes), *meaning* (the significance assigned to a job by an employee based on his/her perceptions and personal values while considering the requirements of the organization or work goal), and *self-determination* (the sense of autonomy an employee has in making his/her own decisions regarding a task without being supervised constantly).

Several studies have revealed that PE fosters employees’ dedication and energy (i.e., engagement) for their jobs (Aggarwal et al., 2020; Towsen et al., 2020; Shams et al., 2021; Juyumaya, 2022). PE, as an employee’s psychological state resulting from empowerment practices at work, is worth paying attention to comprehending the means to elevate the academics’ positive feelings of energy, passion, and enthusiasm (i.e., WE).

At the same time, the traditional employment relationship is weakened, especially in the post-pandemic era (Huang and Chen, 2021; Van der Heijden et al., 2021), where employers and employees are searching for ways to flexibly align their needs. Yet, employees may not want to return to the old collective agreements on working place and time, and the rich variety of individual needs might come to the forefront much more than before (Rudolph et al., 2021). Such a situation supports and encourages idiosyncratic deals (I-deals). I-deals refer to “voluntary, personalized agreements of a non-standard nature negotiated between individual employees and their employers regarding terms that benefit each party” (p. 978) (Rousseau et al., 2006). They are conceptualized as intrinsically motivating because they have been found to have strong relationships with attitudinal and behavioral outcomes (Rosen et al., 2013; Wang and Ma, 2022).

In particular, tasks and work responsibilities (TWR) I-deals, as well as flexibility (FLX) I-deals, have been found to be associated with organizational commitment, personal initiative, WE, and innovative work behavior (Rosen et al., 2013; Kimwolo and Cheruiyot, 2018; Huang and Chen, 2021). TWR I-deals are employment arrangements in which the employee and his or her employer or agent negotiate additional tasks and responsibilities brought to the job; tasks that develop skills, fit personality, and a position that requires unique abilities for the job (Rosen et al., 2013). FLX I-deals, on the other hand, refer to employment arrangements where employees and their managers negotiate work schedules, accommodation of off-the-job demands on assigning duties, completion of work outside the main office, and flexible work times (Rosen et al., 2013). This new and flexible approach to work has often been referred to as “new ways of working” (NWW; Demerouti et al., 2014). The NWW concept includes a wide range of different forms of modern work arrangements and tools, such as working from home, mobile working, having flexible working hours, using videoconferencing, the internet, and other collaborative tools (Blok et al., 2011).

Drawing on a job demands–resources (JD-R) theory-based taxonomy of work characteristics (Crawford et al., 2010; Tims et al., 2012), scholars have elucidated the I-deals–individual outcomes relationships (Hornung et al., 2014; Shams et al., 2021). From the lens of JD-R theory, employees could evaluate the value and significance of environmental factors (such as job resources) in terms of alignment with their personal and professional preferences and goals that subsequently influence their behavior by impacting their psychological representation (Schaufeli and Taris, 2014). In alignment with this argument, there is empirical evidence that indicates PE (a personal resource) as a significant mediating mechanism between I-deals (taken as a unidimensional construct) and WE among academics of higher education (Shams et al., 2021). Nevertheless, the question of whether PE would also mediate the relationship of TWR I-deals and FLX I-deals with WE among academics of the higher institutions is yet to be answered. Thus, this examines the mediating role of PE in the association of TWR I-deals and FLX I-deals with academics’ WE.

At the same time, the studies concerning I-deals call attention to the significance of boundary conditions, particularly the personality characteristics such as the internal locus of control (iLOC) that impacts the effectiveness of I-deals (Ng and Feldman, 2011; Miller, 2015). Scholars have noted that employees with an iLOC take initiatives proactively to improve their environment and situation by searching for and seeking information and knowledge, trying to influence co-workers positively, and attempting to realize better future results (Sharma and Sharma, 2015; Gangai et al., 2016; Alshebami and Seraj, 2022). In this context, it is more likely that employees with a high iLOC, based on their personal and professional needs, would opt for different kinds of I-deals. The JD-R theory also points out that personal resources such as iLOC help an individual to interpret the effectiveness of external environmental factors that affect their behavior at work (Schaufeli and Taris, 2014). Based on this, iLOC as a personality characteristic enhances academics’ WE by increasing the effect of TWR I-deals and FLX I-deals on academics’ PE.

According to JD-R theory, both job resources and personal resources are important drivers of work engagement. This theory further explains that resources do not exist in isolation. Thus, resources, such as TWR I-deals, FLX I-deals, and personal resources such as PE and iLOC, are important in their own way and have an influence on work engagement, but finding the relationship between them and identifying how they interact in work engagement is worth for further investigation. Furthermore, the interaction between job and personal resources, as well as the internal relationship of that interaction, is unknown. Therefore, this study attempts to analyze the WE among academics in higher education from the perspective of job and personal resources. Exploring the mediating and moderating variables underlying this association may advance scholars’ understanding of how and when TWR I-deals and FLX I-deals can be employed in order to promote academics’ WE.

**FIGURE 1:**
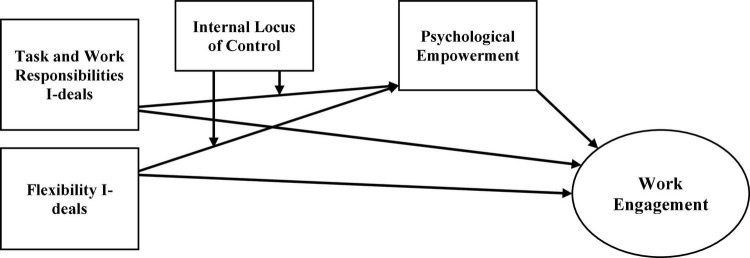
Proposed conceptual model.

In a nutshell, based on JD-R theory, the present study offers an in-depth investigation of the underlying process by which task and work responsibilities (TWR) I-deals and flexibility (FLX) I-deals stimulate academics’ PE and thus influence their WE. Also, this study deliberates on the moderating role of iLOC on the effectiveness of TWR I-deals and FLX I-deals, and academics’ PE in higher education. Furthermore, this study intends to assess a mediation model in which academics’ PE mediates the relationship between TWR I-deals and FLX I-deals and the WE, whereas the iLOC moderates the mediating process. Figure 1 below illustrates the proposed model for this study.

## Theoretical background and hypotheses

### I-deals

Rousseau initially introduced the concept of I-deals in 2001. Generally, I-deals are personalized (non-standardized) employment arrangements negotiated between an employee and the employer (Rousseau, 2001, 2005; Rosen et al., 2013). The initial stream of research on I-deals viewed these work deals from the employment perspective and thus proposed FLX I-deals, reduced workload I-deals, and developmental I-deals (Rousseau and Shperling, 2003). However, scholars such as Hornung et al. (2008) discarded the idea of reduced workload I-deals. Instead, based on the job perspective, task I-deals were proposed in combination with FLX I-deals and developmental I-deals (Hornung et al., 2010). Later on, with the development of this field of research, Rosen et al. (2013) continued improving the structure and theory of I-deals based on the contents and resources represented by different kinds of I-deals. They proposed TWR I-deals by integrating the task I-deals and developmental I-deals in addition to financial incentives I-deals and FLX I-deals. Concerning the financial I-deals, many scholars such as Las Heras et al. (2017) and Marescaux et al. (2019) argue that I-deals related to financial incentives are challenging to observe because recipients tend to refrain from requesting and displaying their financial-related I-deals to others, such as pay raise or compensation packages that are tailored to their unique contributions. Therefore, the present study mainly focuses on TWR I-deals and FLX I-deals.

The TWR I-deals involve additional task activities and responsibilities that are in alignment with employees’ preferences, skills, and strengths—thereby providing growth opportunities to employees and developing their knowledge, skills, and abilities (KSA) to realize a better person-environment (P-E) or person-job (P-J) fit (Rosen et al., 2013; Bal and Dorenbosch, 2015). Past empirical studies have revealed a significant positive correlation of TWR I-deals with the meaning of work (Hornung et al., 2019), innovative performance (Huang and Chen, 2021), innovative work behavior (Kimwolo and Cheruiyot, 2018), and other work-related positive outcomes such as organizational citizenship behavior and work performance (Rofcanin et al., 2016). Similarly, FLX I-deals aim to improve employees’ work–life balance by allowing them to organize their work schedules based on their personal and professional needs. Empirical research has demonstrated a significant positive relationship between FLX I-deals and work–family balance (Bolino and Grant, 2016; Wang et al., 2019). The focus of both I-deals is different in terms of areas (i.e., work and family) and provides different resources to employees. For instance, TWR I-deals provide structural resources to employees that are appropriate for their development and growth. While FLX I-deals mainly focus on the family domain to lessen the loss of an employee’s time, energy, and other non-essential resources.

This study investigates the influence of TWR I-deals and FLX I-deals on the WE among academics in the higher education sector for the following reasons. Nowadays, the increasingly competitive landscape of higher education in national and international arenas has imposed much pressure on academics. In addition to achieving significant performance in research, consultancies, and administration, academics are supposed to shoulder the responsibilities of disseminating knowledge, stimulating critical thinking, mentoring, and encouraging innovation among students, thereby making the academician’s job more complex. On the other hand, the study by Johnsrud and Heck (1998) pointed out that academics tend to enjoy freedom in teaching, research, and the nature of their service. In this situation, negotiating TWR I-deals offers significant autonomy to perform all the aforementioned tasks. For these reasons, TWR I-deals may become more common among HEIs’ academics. Thusly, granting TWR I-deals is more likely to enhance academics’ WE.

Similarly, given the association of work–life balance with academics’ well-being in higher education as reflected in various studies (e.g., Franco et al., 2021) and their emerging desire for work–life balance, FLX I-deals are extremely important in HEIs. In addition, FLX I-deals allow academics to work and teach from home using online platforms such as videoconferencing, the internet, and collaborative tools (Blok et al., 2011; Atiku et al., 2021). Thus, FLX I-deals may help academics perform better in their work–family domain. In addition, similar to TWR I-deals, FLX I-deals are intrinsically motivating and enhance employees’ positive outcomes, such as innovative work behavior (Kimwolo and Cheruiyot, 2018; Huang and Chen, 2021), and hence it is expected to enhance WE—a positive employee outcome.

Given the above discussion, this study investigates the influence of TWR I-deals and FLX I-deals on the academics’ WE.

### I-deals and work engagement

The WE is defined as “a positive, fulfilling, work-related state of mind that is characterized by vigor, dedication, and absorption” (Schaufeli et al., 2002, p. 74). This is the nature of work design that promotes skills, knowledge, and other environmental factors that contribute to developing employees’ positive work-related state of mind. Therefore, in the context of higher education, it is argued that TWR I-deals positively affect the academics’ WE for the following reasons: First, TWR I-deals are intended to improve individuals’ KSA. Academics rely on KSA to manage multiple responsibilities such as teaching, research, and administrative tasks. Thus, academics with higher KSA are more likely to exhibit WE. Second, TWR I-deals indicate that the organization recognizes the value and ability of its employees (Rousseau et al., 2006; Ho and Kong, 2015). In higher education, this recognition can enhance the confidence of academics to be more effective and encourage them to go the extra mile in their work performance by ensuring higher levels of WE. Third, TWR I-deals result in making the work content and characteristics more challenging and autonomous (Hornung et al., 2010); which are positive job attributes that are positively associated with WE (Hornung et al., 2010, 2018). Lastly, TWR I-deals enable individuals to amplify their job resources (such as autonomy) that are theorized to stimulate a motivational process, resulting in positive and active response experiences, such as high performance and WE (e.g., Salanova et al., 2005).

Distinct from TWR I-deals, FLX I-deals are intended to arrange individuals’ work schedules based on their needs, avoid conflict, alleviate any role conflict, attain work–family enrichment, and amplify their work role performance, innovative performance, innovative work behavior, and work efficiency (Las Heras et al., 2017; Hornung et al., 2018; Kimwolo and Cheruiyot, 2018; Wang et al., 2019; Huang and Chen, 2021). The present study argues that FLX I-deals positively affect the WE among academics in higher education for the following reasons. First, as mentioned earlier, academics are involved in multiple tasks with a core focus on teaching and research; all these require concentration, which exhausts their already limited mental and self-control resources (see Halbesleben et al., 2014). When academics assign their time and energy to settle work–family conflict, they may not be able to focus on their work and go the extra mile in performing their job. FLX I-deals can help academics solve problems related to work–family conflict, reduce the stress of distressing non-work-related issues during their working hours, and allocate resources such as attention, passion, time, and positive energy to their work. This offers the best circumstances for academics to foster their WE. Next, FLX I-deals permit academics to have some freedom over their working hours, reasonably allocate their attention, energy, and time, and organize job activities promptly. This helps them to complete their work tasks efficiently and achieve their work goals by ensuring their engagement at work. The foregoing explanation is consistent with the past literature. For instance, the meta-analytic study by Liao et al. (2016) found a significant positive relationship among FLX I-deals and job satisfaction, affective commitment, and continuity at work after retirement. With job satisfaction and affective commitment, academics are likely to be enthusiastically involved in the success of their organizations in any sphere that improve effectiveness, such as through WE. Therefore, this study proposes:

H1a: TWR I-deals positively relate to the academics’ WE.

H1b: FLX I-deals positively relate to the academics’ WE.

### I-deals, psychological empowerment, and work engagement

The motivational process of JD-R theory (Bakker and Demerouti, 2014, 2017) holds that a work environment where job resources are abundantly available against the job demands results in positive outcomes for the employees, like employees’ WE. Job resources have intrinsic and extrinsic motivational potential that serves as a stimulus, making employees feel engaged and energetic, leading to positive outcomes.

I-deals are customized work arrangements that individuals negotiate with their organizations based on their personal and professional needs. More specifically, TWR I-deals are associated with job customization of content such as job duties, responsibilities, and workload.

Indeed, negotiating I-deals enables employees to increase their job resources (e.g., developmental, flexibility, and autonomy at work) (Rousseau, 2005), which increases the meaning of their jobs and improves their WE (Demerouti and Bakker, 2011; Liao et al., 2016; Hornung et al., 2019; Katou et al., 2020).

More specifically, TWR I-deals are associated with increasing job resources through job customization of content such as job duties, responsibilities, and workload (Hornung et al., 2014), while FLX I-deals enable individuals to have choices in scheduling their work, which allows them to gain more resources (e.g., time, energy, attention) and this, as a result, improves their family performance as well as work performance (Bakker and Demerouti, 2014; Las Heras et al., 2017; Erden Bayazit and Bayazit, 2019; Kelly et al., 2020).

Furthermore, besides job resources, personal resources are strong predictors of WE (Halbesleben, 2010). Xanthopoulou et al. (2009) defined personal resources in terms of resiliency, control, and impact on the environment; they may increase engagement and reduce burnout. Since job resources serve an intrinsic motivational role and boost job autonomy and competence (Deci and Ryan, 2000; Bakker and Demerouti, 2007; Van den Broeck et al., 2008). In this context, PE could be considered a personal resource because of its ability to be impacted by different interventions. Furthermore, personal resources have been documented as a mediating mechanism between job resources and WE (Xanthopoulou et al., 2007). Therefore, in higher education, our study argues that an increase in job resources through TWR I-deals and FLX I-deals can enhance academics’ PE by creating a work environment that fosters meaningful work experiences that encourage academics’ sense of competence, self-determination, and an awareness of the influence they have on their work, leading to promoting beneficial outcomes such as WE (Meng and Sun, 2019; Shams et al., 2021).

In a nutshell, TWR I-deals and FLX I-deals offer sufficient autonomy to academics to align their work design with their passions, motivations, and preferences. This autonomy generates a sense of PE among them; consequently, PE will trigger their positive energy, enthusiasm, and dedication (i.e., WE) at work. Thus, we propose:

H2a: PE mediates the relationship between TWR I-deals and the WE among academics.

H2b: PE mediates the relationship between FLX I-deals and the WE among academics.

### Moderating role of internal locus of control

Although I-deals can enhance PE and the WE among academics in higher education, their effectiveness may vary among academics with varying levels of locus of control (LOC). The LOC implies how people perceive or believe that they can control events that can impact them (Rotter, 1966). Based on Rotter’s (1966) classification, the LOC can be internal or external. People with an iLOC (i.e., internals) believe that they are responsible for everything (good or bad) happening in their lives (Levenson, 1974; Akca et al., 2018; Yuwono et al., 2020). Conversely, people with an external LOC (i.e., externals) believe that luck, external forces, or chances are responsible for events in their lives (Dağal and Bayındır, 2017; Yuwono et al., 2020). Strickland (1989) argued that iLOC fosters autonomy, creativity, confidence, and positive initiative among individuals when faced with adverse events and experiences. Hence, the iLOC can be viewed as a source of PE as it predisposes individuals to exert greater effort on work tasks (Spreitzer, 1995; Koberg et al., 1999). Consistent with this, previous studies have shown a significant relationship between iLOC and PE (Ng et al., 2006; Wilson, 2011).

Furthermore, employees who are resilient, i.e., high on iLOC, acquire occupational self-direction or utilize independent judgment, initiatives, and thoughts in their work (i.e., I-deals) (Strümpfer, 1990). Supporting the notion, Ng and Feldman (2011) argued that “employees with a high internal locus of control are significantly more likely to obtain idiosyncratic employment deals for themselves” (p. 186). Thus, given the significance of iLOC in enhancing PE and the need for being independent at work in terms of judgment, initiatives, and thoughts, it is more likely that the academics’ PE in higher education will be enhanced if they are granted different types of I-deals based on the personal and professional requirements.

In alignment with the JD-R theory, which postulates that human behavior results from an interaction between personal (personal characteristics) and environmental factors (i.e., job resources) (Schaufeli and Taris, 2014). Along with somewhat similar lines, Judge et al. (2000) argued that an employee’s personality characteristics (such as LOC) determine how he/she perceives their job characteristics would impact job performance and satisfaction. Besides, empirical evidence is available that demonstrates a significant positive relationship between iLOC with subjective well-being (Dave et al., 2011) and PE (e.g., Wang et al., 2013; Shirvan, 2019). Thus, there is a possibility that academics with a high iLOC would prefer I-deals based on their personal and professional needs, which would enhance their PE.

Notably, the TWR I-deals enable employees to increase their job resources by customizing the work content that they are interested in doing and are very good at. In alignment with this, Spreitzer (1995) and Li et al. (2015) argued that in an environment where the job is meaningful, competence is recognized, colleagues support them, and more freedom is given to make decisions and respond to problems in their way immediately, employees with an iLOC feel more psychologically empowered, resulting in higher performance (Li et al., 2015). Thus, TWR I-deals offer skill development opportunities to academics with an iLOC on the one hand, and, on the other hand, it is more likely to enhance their PE.

Likewise, with the iLOC, academics strive to increase their job resources, such as time and energy, to perform well in their family domain. FLX I-deals allow academics to schedule their time based on their personal needs to a certain extent, helping them toward work–family enrichment (Tang and Hornung, 2015) and creating a pleasant work environment by coping with job demands that create strain (Hornung et al., 2014). Upon obtaining FLX I-deals, individuals with a high iLOC think, feel, and behave positively and favorably toward an organization (Qurrahtulain et al., 2020), thereby enhancing their PE. Thus, we propose:

H3a: ILOC moderates the positive relation between TWR I-deals and PE, such that the positive relation is stronger for academics with a high iLOC than for academics with a lower iLOC.

H3b: ILOC moderates the positive relation between FLX I-deals and PE, such that the positive relation is stronger for academics with a high iLOC than for academics with a lower iLOC.

At this stage, this study proposes the effect of I-deals on the WE among academics in higher education, and it investigates the mediating role of PE and the moderating role of iLOC. This study further argues that iLOC may enhance the indirect effects of I-deals on the WE among academics through their PE. Studies have shown that individuals with a high iLOC exhibit a higher level of WE (Sharma and Sharma, 2015; Singh et al., 2020). These studies have demonstrated that individuals with iLOC often try to take charge of the situation themselves and engage themselves in work that gives them satisfaction.

According to JD-R theory (Schaufeli and Taris, 2014), the iLOC is one of the personal resources. Also, past studies have supported the moderating role of personal resources in the association between adverse working conditions and well-being (Mäkikangas and Kinnunen, 2003; Pierce and Gardner, 2004; Schaufeli and Taris, 2014). We argue that TWR I-deals and FLX I-deals, when interacting with iLOC, may improve the relationship between TWR I-deals, FLX I-deals, and PE. Although TWR I-deals and FLX I-deals provide a basis for improving academics’ WE; however, such benefits cannot be effectively realized unless personality factors (i.e., iLOC) interact with them. Furthermore, the presence of iLOC enhances the academics’ PE and WE. Therefore, higher iLOC enlarges the benefits of TWR I-deals and FLX I-deals by strengthening academics’ PE. Specifically, we suggest that the more there is a high level of iLOC, the more it will strengthen the indirect relationship of PE between TWR I-deals, FLX I-deals, and WE. Based on the above discussion, by combining the aforementioned hypotheses, this study proposes the following moderated-mediating effect hypotheses:

H4a: ILOC moderates the mediating effect of PE on the relationship between TWR I-deals and the WE among academics, such that the mediating effect is stronger for academics with a high iLOC.

H4b: ILOC moderates the mediating effect of PE on the relationship between FLX I-deals and the WE among academics, such that the mediating effect is stronger for academics with a high iLOC.

## Materials and methods

### Sampling

For this study, hypotheses were tested using data collected from the academics working in the sixteen large-sized HEIs in Pakistan (as per the HEC, 2021 website, 2017–2018). The large-sized HEIs were selected for two main reasons: First, large-sized HEIs offer several academic programs and thus have a significant number of faculty members (academics). Second, in their study, Khan and Yusoff (2016) underlined that academics within large-sized HEIs suffer from a broad range of work stressors that eventually result in lower work engagement.

### Procedure

An e-mail survey was used to collect the data. According to Heerwegh (2009), the e-mail survey format allows survey participants to respond at their convenience, assists in accessing a geographically dispersed population, and minimizes social desirability bias. Given the fact that, besides being geographically dispersed, academics typically work in an environment characterized by high job demands with very tight work schedules, the e-mail survey method was deemed appropriate for collecting high-quality, relatively unbiased data. In total, 650 academics were emailed an online survey; 329 responses, representing a response rate of 50.6%, were received. This response rate is considered highly acceptable, as a response rate of 11% or less is considered reasonable for e-mail surveys (Saunders, 2012).

### Respondents’ characteristics

Of the 329 respondents, 87% were men, whereas 13% were women. In total, 35% of the respondents held a Ph.D., while the remaining 65 held an MS/MPhil. Concerning designation, 27% of the total respondents were full professors, 8% were associate professors, 52% were assistant professors, and 13% were lecturers. With regard to work experience, out of the total, 3% had 4–6 years of work experience, 11% had 7–9 years of work experience, and 38% had 10–12 years of work experience, while 48% of the respondents had more than 12 years of teaching experience. This study noted 42.5 as the average age of the respondents.

### Measures

The survey used in this study comprised five sections. The first section contained questions to inquire about demographic information. However, the survey’s second, third, fourth, and fifth sections included items related to WE, TWR I-deals, FLX I-deals, PE, and iLOC constructs, respectively. Except for the WE construct; all the aforementioned constructs were measured on a 5-point Likert scale ranging from “1” = “strongly disagree” to “5” = “strongly agree.” However, the WE construct was assessed on a 7-point Likert scale, which ranged from “never = 1” to “always = 7.”

#### Work engagement

This study employed a nine-item “Utrecht Work Engagement Scale (UWES)” to measure the three dimensions of WE (Schaufeli et al., 2006). The coefficient value of this instrument in this study was 0.952. A sample item was “My job inspires me.”

#### I-deals

This study used Rosen et al.’s (2013) scale for assessing both TWR I-deals and FlX I-deals constructs. The TWR I-deals construct is comprised of six items (α = 0.914). “I have successfully asked for extra responsibilities that take advantage of the skills that I bring to the job” was one of the sample items to measure TWR I-deals. The FLX I-deals was measured with three items (α = 0.811). “My supervisor considers my personal needs when making my work schedule” represented one of the items to measure FLX I-deals.

#### Psychological empowerment

A four-dimensional 12-item scale called the ‘Psychological Empowerment Instrument (PEI)’ by Spreitzer (1995) was adopted to measure the PE construct. The coefficient reliability value of this instrument was 0.908. A sample item was, “I am confident about my ability to do my job.”

#### Internal locus of control

The present study adopted Levenson’s (1981) eight-item scale to measure the iLOC construct. The coefficient reliability value of this instrument was 0.928. A sample item was, “My life is determined by my own actions.”

### Strategy of analysis

In this study, the analysis of the data was performed by utilizing a variance-based structural equation modeling (PLS-SEM) that provides more reliable construct scores in comparison with the covariance-based structural equation modeling (PLS-CB) (Henseler et al., 2015). In addition, for complex models and explanatory research, PLS-SEM is considered a perfect fit (Henseler et al., 2018; Ringle et al., 2020). Given the nature of this study to derive managerial recommendations, i.e., explanatory and prediction and the use of complex models (including direct, mediating, and moderating effects), this study applied PLS-SEM. Following the suggestion of Hair et al. (2019), the PLS-SEM analysis involves a two-step procedure, i.e., the assessment of the measurement model and the structural model. Also, we examine the mediating effect of PE and the moderating effect of iLOC by the SMART-PLS program (Ringle et al., 2015). In order to examine the simple slopes of those significant moderation results, we used PROCESS for SPSS v24 (Hayes, 2017).

## Results

### Statistical analysis

Prior to data analysis, this study performed data screening to probe the identification of missing values, assessment of outliers, test of normality, and examine common method bias (CMB). Since it was mandatory to answer all questions in the survey; therefore, there was no issue of missing value in this study. The data in the present study were assessed for detecting the univariate *via* a standardized (Hair et al., 2010) and multivariate outliers utilizing a Mahalanobis distance test (Tabachnick and Fidell, 2013) with a threshold value of ±4 in the SPSS version 24. Following the rule of thumb, we identified five multivariate outliers (Hair et al., 2010), which were removed accordingly. Thus, this study considered 324 responses for analysis purposes.

Furthermore, the normality of data in the present study was assessed through skewness and kurtosis criteria (Hair et al., 2017). According to DeCarlo (1997), if the skewness and kurtosis values vary from –3 to +3, it indicates that the data are normal. Based on the evidence presented in Table 1, the skewness values varied from –1.155 to 0.400, whereas the kurtosis values varied from –1.101 to 1.587, indicating that the data are normal in this study.

**TABLE 1:** Test of normality and Fornell–Larcker criterion (*N* = 324).

	Skewness		Kurtosis		1	2	3	4	5
Constructs	Statistic	*SE*	Statistic	*SE*					
FLX I-deals	–0.880	0.135	1.508	270	0.852				
PE	–1.110	0.135	1.587	270	0.495	0.724			
TWR I-deals	–0.839	0.135	0.823	270	0.617	0.477	0.836		
WE	–1.155	0.135	1.522	270	0.418	0.385	0.507	0.851	
iLOC	0.400	0.135	–1.101	270	–0.411	–0.646	–0.646	–0.288	0.814

FLX I-deals, flexibility I-deals; PE, psychological empowerment; TWR I-deals, task and work responsibilities I-deals; WE, work engagement; iLOC, internal locus of control; SE, standard error.

Additionally, the mean values, standard deviation values, and correlation among study variables are portrayed in Table 2. With regard to mean values, Sekaran and Bougie (2016) suggested that, on a 5-point Likert Scale, mean values equal to or less than 2.99 are considered low, 3–3.99 represent moderate, and mean values greater than 4 are regarded as high. While on a seven-point Likert scale, values that are less than 4.99, between 5 and 5.99, and above 6 are deemed low, moderate, and high, respectively. Based on Table 2, the mean values of TWR I-deal, FLX I-deals, PE, and iLOC are between 3.068 and 3.893, indicating a moderate level of academics’ perception of the presence of these elements in the HEIs. Similarly, the mean value of the WE construct, which is 5.456, indicates that academics in the HEIs are moderately engaged in their jobs (refer to Table 2 below).

**TABLE 2:** Descriptive statistics and correlation analysis (*N* = 324).

Constructs	Mean	*SD*	1	2	3	4	5
FLX I-deals	3.713	0.854	1				
PE	3.812	0.779	0.510**	1			
TWR I-deals	3.892	0.929	0.604**	0.496**	1		
WE	5.456	1.325	0.411**	0.396**	0.493**	1	
iLOC	3.068	0.982	–0.400**	–0.211**	–0.627**	–0.273**	1

**p < 0.01. FLX I-deals, flexibility I-deals; PE, psychological empowerment; TWR I-deals, task and work responsibilities I-deals; WE, work engagement; iLOC, internal locus of control; SD, standard deviation.

Furthermore, concerning the correlation between study variables, as shown in Table 2, TWR I-deals are positively correlated with PE and WE (*r* = 0.496, *p* < 0.01 and *r* = 0.493, *p* < 0.01, respectively). FLX I-deals are positively correlated with PE and WE (*r* = 0.510, *p* < 0.01 and *r* = 0.411, *p* < 0.01, respectively). The correlation between PE and WE is significantly positively correlated (*r* = 0.396, *p* < 0.01). On the contrary, the results presented in Table 2 show that the correlation between iLOC as a moderator and other study constructs such as TWR I-deals, FLX I-deals, PE, and WE is significantly negatively correlated (i.e., *r* = –0.627; *r* = –0.40; *r* = –0.21; and *r* = –0.273, respectively). However, the coefficient values of the interaction term TWR I-deals*iLOC and FLX I-deals*iLOC with PE (i.e., β = 0.194 and β = 0.025, respectively) and further between TWR I-deals*iLOC and FLX I-deals*iLOC with WE through PE (β = 0.030 and β = 0.004, respectively) are positive, indicating that the iLOC moderates the relationship between this study’s constructs (refer to Table 3).

**TABLE 3:** Hypotheses testing.

					Coefficients	*SD*	LLCI	ULCI	T-value	*p*-value
TWR I-deals	→	WE			0.415	0.055	0.301	0.519	7.438	0.000
FLX I-deals	→	WE			0.161	0.059	0.051	0.272	2.700	0.007
TWR I-deals	→	PE			0.359	0.082	0.203	0.516	4.383	0.000
FLX I-deals	→	PE			0.261	0.061	0.144	0.383	4.279	0.000
PE	→	WE			0.155	0.042	0.075	0.241	3.689	0.000
TWR I-deals	→	PE	→	WE	0.056	0.021	0.024	0.110	2.634	0.008
FLX I-deals	→	PE	→	WE	0.041	0.015	0.018	0.076	2.752	0.006
TWR I-deals*iLOC	→	PE			0.194	0.041	0.050	0.338	2.121	0.018
FLX I-deals*iLOC	→	PE			0.025	0.716	–0.143	0.4046	0.111	0.370
TWR I-deals*iLOC	→	PE	→	WE	0.030	0.015	0.007	0.057	1.964	0.025
FLX I-deals*iLOC	→	PE	→	WE	0.004	0.011	–0.011	0.026	0.346	0.365

FLX I-deals, flexibility I-deals; PE, psychological empowerment; TWR I-deals, task and work responsibilities I-deals; WE, work engagement; iLOC, internal locus of control; SD, standard deviation; LLCI, lower limit confidence interval; ULCI, upper limit confidence interval.

### Common method bias

As the data collection for this study was from a single source, Kock’s (2015) guidelines were considered to make sure that the data were free from CMB. Kock (2015) suggested applying the full collinearity approach to assess the CMB. Assessing the full collinearity requires regressing all the study variables against a common variable; and if the value of VIF is equal to or less than 5, then it indicates no bias in the single-source data. As evident from Table 4, the analysis resulted in VIF values of less than five. Hence, there was no issue of bias from a single source with the data in this study.

**TABLE 4:** Assessment of full collinearity.

TWR I-deals	FLX I-deals	PE	iLOC	WE
2.611	1.769	1.540	1.701	1.395

Flx I-deals, flexibility I-deals; PE, psychological empowerment; TWR I-deals, task and work responsibilities I-deals; WE, work engagement; iLOC, internal locus of control.

### PLS-SEM path modeling

#### Measurement model

In this study, we followed the suggestions by Hair et al. (2019) for the assessment of the measurement model. The assessment of the measurement model is performed through internal consistency and construct validity. According to Sekaran and Bougie (2016), construct validity entails convergent and discriminant validity. Convergent validity is established when the average values extracted (AVE) are equal to or greater than 0.50, and the factor loadings for a specific construct are equal to or higher than 0.708 or 0.70 (Hair et al., 2017). However, factor loadings that range between 0.50 and 0.70 are also deemed acceptable (Chin, 1998). Moreover, Hair et al. (2017) suggested deleting items with loading values of less than 0.40. Since the AVE value of the PE construct was lower than the standard criterion, one item of PE (i.e., PE10) was accordingly removed.

On the contrary, Hair et al. (2017) have recommended that if the composite reliability (CR) value or the values of AVE are not affected, then the item values ranging between 0.40 and 0.70 are not deleted. Table 5 displays that the measurement items’ values fall within the range as per the standard criteria. The constructs’ AVE and CR values in the present study fulfilled the passing criteria; therefore, the items with factor loadings < 0.70 have been retained. As values of all constructs are within the acceptable range of standard criteria (see Table 5), this confirms adequate convergent validity. This study applied the Fornell–Larcker criterion for establishing discriminant validity. According to this criterion, the square root of all constructs’ AVEs should be higher than their correlation with the respective constructs in the model. The results presented in Table 1 above confirm the presence of discriminant validity in this study.

**TABLE 5:** Convergent validity.

Constructs	Items	Factor loadings	AVE	CR
Work engagement	WE1	0.688	0.725	0.959
	WE2	0.889		
	WE3	0.844		
	WE4	0.865		
	WE5	0.842		
	WE6	0.875		
	WE7	0.824		
	WE8	0.897		
	WE9	0.893		
Task and responsibilities I-deals	TWR1	0.837	0.700	0.933
	TWR2	0.875		
	TWR3	0.890		
	TWR4	0.835		
	TWR5	0.816		
	TWR6	0.759		
Flexibility I-deals	FLX1	0.863	0.726	0.888
	FLX2	0.853		
	FLX3	0.840		
Psychological empowerment	PE1	0.589	0.524	0.923
	PE2	0.809		
	PE3	0.704		
	PE4	0.761		
	PE5	0.722		
	PE6	0.754		
	PE7	0.824		
	PE8	0.792		
	PE9	0.688		
	PE10	Deleted		
	PE11	0.663		
	PE12	0.621		
Internal locus of control	iLOC1	0.859	0.663	0.940
	iLOC2	0.892		
	iLOC3	0.803		
	iLOC4	0.873		
	iLOC5	0.740		
	iLOC6	0.865		
	iLOC7	0.770		
	iLOC8	0.688		

AVE, average variance extracted; CR, composite reliability.

#### Structural model

This study followed Hair et al.’s (2019) recommendations by applying 5,000 resample bootstrapping procedures for testing hypotheses. Following the suggestion by Hahn and Ang (2017), *p*-values, *t*-values, and confidence intervals were used to conclude the significant results of the hypotheses.

Table 3 displays that the relationship between TWR I-deals and WE among academics is significantly positive (β = 0.415, *t* = 7.438, *p* < 0.05), validating hypothesis H1a. In the same way, this study discovered a significant positive relationship of FLX I-deals with WE (β = 0.161, *t* = 2.700, *p* < 0.05), supporting hypothesis H1b.

It has been documented that the influence of PE on the WE is significantly positive (β = 0.155, *t* = 3.689, *p* < 0.05). Table 5 results confirm PE’s mediating role in the relationship between TWR I-deals and WE (β = 0.056, *t* = 2.634, *p* < 0.05) and FLX I-deals and WE (β = 0.041, *t* = 2.752, *p* < 0.05). Therefore, both hypotheses H2a and H2b are accepted. In addition, to assess the mediation type, the significance of the direct and indirect effects was checked. As results for both direct and indirect effects were significant and in the same direction (refer to Table 7); it can be considered a complementary type of partial mediation (Nitzl et al., 2016; Hair et al., 2017).

**TABLE 6:** Significance analysis of the direct and indirect effects and mediation type.

Structural path			Direct effect	CI direct effect		*T*-value	*P*-value	Indirect effect	CI indirect effect		*T*-value	*P*-value	Mediation type
				LLCI	ULCI				LLCI	ULCI			
TWR I-deals	→	WE	0.415	0.319	0.502	7.708	0.000**	0.056	0.027	0.089	2.817	0.003**	Partial mediation (Complementary)
FLX I-deals	→	WE	0.161	0.072	0.254	4.458	0.000**	0.040	0.020	0.067	2.800	0.003**	

FLX I-deals, flexibility I-deals; TWR I-deals, task and work responsibilities I-deals; WE, work engagement; LLCI, lower limit confidence interval; ULCI, upper limit confidence interval. **P < 0.01.

**TABLE 7:** Conditional indirect effect results.

Moderator value	Conditional indirect effect	Bootstrap *SE*	Bootstrap LLCI	Bootstrap ULCI
Outcome variable: WE; Independent variable: Task and work responsibilities I-deals				
2.0000	0.0723	0.0314	0.0195	0.1423
3.0000	0.1445	0.0354	0.0827	0.2206
4.2500	0.2348	0.0527	0.1360	0.3505
Outcome variable: WE; Independent variable: Flexibility I-deals				
2.0864	0.0629	0.0381	–0.0112	0.1397
3.0687	0.1661	0.0403	0.0915	0.2471
4.0510	0.2693	0.0555	0.1635	0.3835

LLCI, lower level confidence interval; ULCI, upper level confidence interval, level of confidence = 95%, number of bootstrap samples = 5,000; SE, standard error.

**GRAPH 1:**
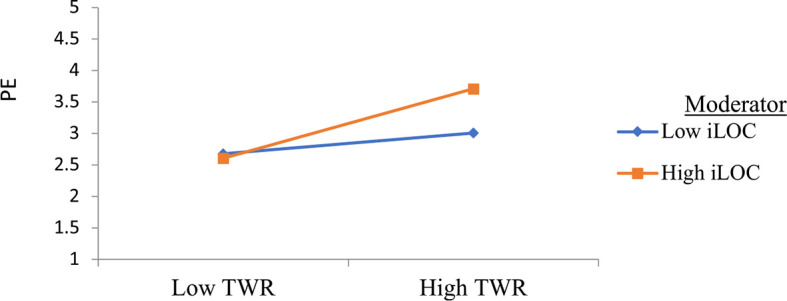
Interaction plot of internal locus of control and task and work responsibilities I-deals.

Based on hypotheses H3a and H3b, it was expected that the iLOC would moderate the association of TWR I-deals and FLX I-deals with PE. Furthermore, supporting the moderation hypotheses, the strength of mediation, i.e., PE (indirect value), is expected to depend on the value of moderation (i.e., iLOC), indicating a conditional in-direct effect or moderated-mediation (Hayes and Rockwood, 2020). Following Hair et al.’s (2019) recommendation, the moderating effect of the iLOC was examined *via* a two-stage approach. Based on the results presented in Table 5 above, hypothesis H3a is supported because the coefficient of interaction (between TWR I-deals and iLOC) has a significant effect on the PE (β = 0.194, *t* = 2.358, *p* < 0.05), thereby, supporting H3a. Furthermore, it was found that a higher value of iLOC would result in a stronger relationship between TWR I-deals and PE. Graph 1 illustrates the moderating impact of iLOC on the association of TWR I-deals with PE. Contrary to our expectation, the present study, however, did not confirm the moderating effect of iLOC on the association of FLX I-deals and PE (β = 0.025, *t* = 0.111, *p* > 0.05), rejecting H3b.

Finally, to test the last two hypotheses of this study, i.e., assessing the impact of moderated-mediation on the TWR I-deals-WE relationship and FLX-WE relationship, we employed Hayes’s (2017) PROCESS macro of SPSS. Table 3 shows that the estimates’ values, bootstrap SE, and bootstrap LLCI and ULCI for the conditional indirect effects of TWR I-deals, respectively, fall at the low, medium, and high levels of iLOC. Results in Table 3 indicate that the conditional indirect effect of TWR I-deals is significantly stronger at the higher level of iLOC (0.2348) and significant with relatively lesser strength at the lower level of iLOC (0.0723). Therefore, hypothesis H4a is supported.

Furthermore, concerning the moderated-mediation impact of the FLX I-deals–WE relationship, results indicate that the conditional indirect effect of FLX I-deals is not significant at the low level of iLOC (value of estimate = 0.0381, 95% CI = [-0.0112, 0.1397], involving zero). However, with the increase in value of iLOC, the difference effect is significant (value of estimate = 0.0555, 95% CI = [0.1635, 0.3835], without zero), demonstrating support for H4b.

## Discussion

The extant literature illustrates that for HEIs, being complex and knowledge-intensive organizations, achieving superior performance and sustainable competitive advantage mainly depends on the commitment and engagement of their academics (Nazir and Islam, 2017; Aboramadan et al., 2019; Shrand and Ronnie, 2021). Relatedly, in the pursuit of enhancing academics’ WE, previous studies have emphasized the role of I-deals (Rosen et al., 2013; Shams et al., 2021). Despite the significance of I-deals in resulting in positive work-related outcomes and their potential for enhancing employer–employee relationships, the volume of literature on I-deals is relatively thin (Van der Heijden et al., 2021). Thus, given the importance, this study, by employing the JD-R theory, investigated the role of two types of I-deals, i.e., TWR I-deals and FLX I-deals on WE. In addition, this study also probed the role of iLOC as a moderator in the indirect association of TWR I-deals and FLX I-deals with WE in the higher education sector through PE.

This study found a significant positive relationship between TWR I-deals and WE, providing support to the first hypothesis (H1a). In alignment with the previous studies, this result explains that granting TWR I-deals results in making the job content and characteristics more challenging and autonomous (Hornung et al., 2010); challenging work and job autonomy are positively associated with WE (Hornung et al., 2010, 2018). Similarly, this study hypothesized a significant positive relationship between FLX I-deals and WE. The finding confirmed the FLX I-deals–WE relationship, which substantiates the second hypothesis (H1b) of this study. This result illustrates that FLX I-deals allow academics to arrange work schedules, accommodate off-the-job demands on their assigned duties, flexible work times, and complete work outside the main office by applying videoconferencing, internet, and collaborative tools (NWW; Rosen et al., 2013; Demerouti et al., 2014; Atiku et al., 2021).

By doing this, they can avoid role conflicts, improve their work–life balance, and increase their work efficiency, work role performance, and innovative performance (Las Heras et al., 2017; Hornung et al., 2018; Kimwolo and Cheruiyot, 2018; Wang et al., 2019; Huang and Chen, 2021), as well as WE. Furthermore, this result has more practical implications for academics in the HEIs, especially after the post-pandemic era (Atiku et al., 2021). From the lens of JD-R theory, TWR I-deals and FLX I-deals allow academics to increase their job resources (e.g., developmental, autonomy, and flexibility at work). The availability of these job resources makes academics’ jobs meaningful and enhances their engagement at work (Demerouti and Bakker, 2011).

Next, we examined the mediating role of PE in the relationship of TWR I-deals and FLX I-deals with WE. This study found that PE mediated the TWR I-deals–WE and FLX I-deals–WE relationships, substantiating the third and fourth hypotheses (i.e., H2a and H2b). Consistent with these findings, the authors have submitted that I-deals engender a work environment that nurtures academics’ meaningful work experiences and encourages their self-determination, sense of competence, and an awareness of the influence they have on their work (i.e., dimensions of PE), leading to promoting beneficial outcomes such as WE (Meng and Sun, 2019; Shams et al., 2021). These results are consistent with JD-R theory, which postulates that personal resources (i.e., PE) not only carry the effect of job resources on WE but that personal resources such as PE also mediate the job resources (TWR I-deals and FLX I-deals) — WE relationship (Xanthopoulou et al., 2009).

In addition to mediation, this study hypothesized two hypotheses (i.e., H3a and H3b) to assess the moderating role of iLOC on the association of TWR I-deals and FLX I-deals with PE. This study found that iLOC as a personal characteristic can boost the positive influence of TWR I-deals on PE, hence substantiating H3a. The present empirical evidence of iLOC as a moderator is consistent with the JD-R theory’s perspective. However, the moderating role of iLOC on the association of FLX I-deals with PE was found to be insignificant, rejecting H3b. Although previous studies have confirmed the presence of a moderator in the relationship between I-deals and PE (e.g., Miller, 2015). However, contrary to our expectations, the moderating influence of the iLOC on the FLX I-deals–PE relationship was not significant. The reason may be that academics in higher education, whether they have high or low iLOC, have a robust aspiration for work–life balance and value this resource, so their iLOC did not have a significant moderating influence on the FLX I-deal–PE relationship.

Finally, we examined the last two proposed hypotheses (H4a and H4b) to assess the moderating role of iLOC (a personal resource) on the indirect association of TWR I-deals and FLX I-deals with WE through PE. This study found that iLOC positively moderated the indirect influence of TWR I-deals on the WE among academics *via* PE, providing support for H4a. This result is consistent with previous studies by indicating that individuals with a high iLOC often try to take control of the environment themselves and engross themselves in work, thereby deriving satisfaction (Sharma and Sharma, 2015; Singh et al., 2020), which as a result leads to PE and a higher level of WE (Shams et al., 2021). Similarly, this study also confirmed the moderating role of iLOC on the indirect influence of FLX I-deals on the WE among academics *via* PE. In alignment with previous studies, this finding indicates that negotiating and granting FLX I-deals to academics with a high iLOC instills a sense of organizational favor and trust in them (Wang and Long, 2018). Consequently, they think, feel, and behave positively and favorably for their organization (Qurrahtulain et al., 2020), thereby enhancing their PE. This PE as a personal resource fosters academics’ WE (Shams et al., 2021; Juyumaya, 2022). In alignment with the JD-R theory, personal resources such as iLOC help an individual to interpret the effectiveness of external environmental factors (such as TWR I-deals and FLX I-deals) that subsequently affect their behavior at work (i.e., WE) (Schaufeli and Taris, 2014).

### Theoretical implications

The present study offers the following theoretical implications: First, this study further provides empirical evidence of the influence of TWR I-deals on academics’ WE, which supplements related research on the influence of TWR I-deals on WE among academics in higher education in the context of Pakistan. In addition, the present study also demonstrates that FLX I-deals can foster academics’ WE, though the fostering impact is lower than TWR I-deals. Still, this is a pioneering study that provides empirical evidence that FLX I-deals can foster the WE among academics of higher education to some extent, making up for the dearth of studies on the prevailing FLX I-deals in the working environment. The review of the extant literature reveals that only Las Heras et al.’s (2017) study exhibits that FLX I-deals can improve work performance by improving family performance.

Second, PE, which represents how empowered an individual feels, is vital to promoting WE among academics. Based on the JD-R theory, this study deliberates on how an increase in job resources through granting TWR I-deals and FLX I-deals can increase academics’ personal resources (i.e., PE) and consequently affect their WE. In simple words, TWR I-deals and FLX I-deals serve as means for academics to increase their job resources to make the job meaningful and achieve P-J fit or P-E fit. Thus, negotiating and obtaining I-deals enhance their PE, which, in turn, influence their WE. This finding explores the psychological mechanism that relates TWR I-deals and FLX I-deals with desirable work outcomes such as WE.

Third, I-deals are more costly than standardized work arrangements (Lee et al., 2015). Therefore, personality factors such as iLOC should be taken into consideration while scrutinizing the I-deals’ mechanism. Considering personality factors helps explain the types of people who could benefit more from I-deals. We selected the iLOC as the moderating variable by integrating the existing theoretical basis and practical background information on personality characteristics. This study concluded that when the iLOC is higher, TWR I-deals have a stronger impact on PE and WE among academics in higher education.

Similarly, this study also found that the iLOC magnifies the effectiveness of FLX I-deals in enhancing academics’ WE through their PE. Although FLX I-deals provide a basis for enhancing academics’ WE, such a benefit cannot be effectively realized unless a personality factor (i.e., iLOC) positively interacts with it. Lastly, this study makes the JD-R theory even more useful by answering the research call of scholars to examine the personality factors influencing I-deals of different kinds.

### Practical implications

Given the dire need, especially in higher education, to attract and maintain engaged academics to perform well and stay competitive nationally and globally, our study shows that TWR I-deals and FLX I-deals can enhance academics’ WE. Hence, they are of great value to practitioners in HEIs.

More specifically, TWR I-deals provide structural resources to academics that are appropriate for their growth and development. Similarly, TWR I-deals offer significant job autonomy by allowing academics to customize their job content in a way that allows them to perform their tasks related to teaching, research, consultation, and administrative work more effectively.

Flexibility I-deals, on the other hand, mainly focus on the family domain to lessen the loss of academics’ time, energy, and other non-essential resources. FLX I-deals provide academics with maximal autonomy in carrying out their work by enabling them to negotiate their work schedules, accommodate off-the-job demands on assigning duties, complete work outside the main office, and work flexible hours. Academics could carry out their work tasks and teach through online teaching platforms. Especially, the post-pandemic creates much more significance in using FLX I-deals in the HEIs (Atiku et al., 2021). These I-deals allow academics to achieve a better work–life balance and perform better in their work domain.

In a nutshell, granting specific I-deals based on personal and professional needs elevates academics’ enthusiasm, passion, and positive feelings of energy at work (i.e., WE). Moreover, the mean value of TWR I-deals (i.e., 3.892) and FLX I-deals (i.e., 3.713) endorses the presence of these I-deals in the HEIs. Furthermore, given the empirical support for the relationship between TWR I-deals and FLX I-deals and WE, institutionalization and systematization of TWR I-deals and FLX I-deals in HEIs will aid in motivating and attracting better talents.

In addition to fostering academics’ WE, this study found that TWR-Ideals and FLX I-deals also promote academics’ PE. Furthermore, PE partially mediated the relationship of TWR I-deals and FLX I-deals with WE. These findings imply that a portion of the effect of TWR I-deals and FLX I-deals on WE is mediated through PE, whereas TWR I-deals and FLX I-deals still explain a portion of WE that is independent of PE. Thus, both TWR I-deals and FLX I-deals are essential elements in promoting the PE of HEIs’ academics, and PE subsequently enhances their engagement at work. Therefore, leaders and decision-makers in higher education should consider TWR I-deals and FLX I-deals to enhance both PE and WE. Consistent with this, the top management of HEIs should devise work-related policies and create an environment in which academics feel encouraged to propose TWR I-deals or FLX I-deals, and in reaching these I-deals, the manager or supervisor of the respective HEI should play an active role.

Lastly, this study offers essential instruction to the top management of HEIs on ways to employ TWR I-deals and FLX I-deals. In particular, this research indicates that iLOC impacts the effectiveness of both TWR I-deals and FLX I-deals. With a high iLOC, TWR I-deals and FLX I-deals have a stronger effect on focal academics’ PE and the WE. Thus, when negotiating TWR I-deals and FLX I-deals with academics, the manager or supervisor should take into account the personality characteristics, i.e., iLOC, of the academics and carefully consider whether they agree to those I-deals to improve the usefulness of TWR I-deals and FLX I-deals.

### Limitations and future research directions

Similar to other studies, this research is no exception and has the following limitations: First, this study used single-source data for analysis, where only the academics responded to the questions used in the survey, leading to a potential problem of CMB. In the future, researchers can collect data, especially while measuring the academics’ WE from multiple participants (such as the dean/head of department/supervisor) separately, to minimize measurement errors. Second, the cross-sectional research design is another limitation of this study. As a cross-sectional study lacks generalizability as well as the ability to assess causal relationships; therefore, future studies could use longitudinal research design to strengthen the rigor of this study’s findings. Longitudinal designs collect data from multiple time points, which may increase the interpretability of the results and allow stronger causal conclusions to be made about the constructs. Third, the focus of this study has been on exploring the mechanisms of TWR I-deals and FLX I-deals for the academics of the large-sized HEIs of Pakistan. Hence, the representation might not be sufficient. Future studies could expand this study’s score to other medium- and small-sized HEIs to increase samples’ representativeness and diversity. Lastly, this research mainly focused on the mechanism of TWR I-deals and FLX I-deals from the personality factor (iLOC) perspective. However, the effects of these I-deals may be influenced by many other situational factors, such as age and organizational policies. In the future, researchers could take into account the situational factors to comprehend the I-deals mechanism.

## Conclusion

We inferred in our study that TWR I-deals and FLX I-deals are of crucial importance for ensuring WE among academics, while acceptance of study hypotheses shows the significance of TWR I-deals and FLX I-deals in fostering WE through iLOC. The iLOC moderates the indirect influence of TWR I-deals on academics’ WE *via* PE. Therefore, TWR I-deals and FLX I-deals, iLOC, and PE are essential for HEIs to maintain a team of engaged academics. Moreover, the limitations and implications of our study provide an opportunity for future research in the same domain.

## Data availability statement

The original contributions presented in this study are included in the article/supplementary material, further inquiries can be directed to the corresponding author.

## Author contributions

All authors listed have made a substantial, direct, and intellectual contribution to the work, and approved it for publication.
